# Skills and Resources of Psychiatric Mental Health Nurses to Support a Long and Uncertain Recovery Journey: A Grounded Theory Approach

**DOI:** 10.1111/jpm.70071

**Published:** 2025-12-02

**Authors:** Ryo Odachi, Hironori Yada, Keiichiro Adachi, Momoko Buyo

**Affiliations:** ^1^ Division of Health Sciences, Graduate School of Medicine The University of Osaka Suita Japan; ^2^ Faculty of Human Health Sciences Shunan University Shunan Japan; ^3^ Faculty of Health Sciences Yamaguchi University Graduate School of Medicine Ube City Japan

**Keywords:** long‐term care, mental health services, persons with psychiatric disorders, psychiatric nursing, uncertainty

## Abstract

**Introduction:**

The importance of recovery‐focused practice is widely recognised. However, limited attention has been paid to how nurses continue to engage reflectively and creatively in long‐term care settings.

**Aim:**

This study explored how nurses provided recovery‐oriented care for people with mental illness in long‐term, complex, and uncertain care settings.

**Method:**

A grounded theory approach was used to analyse data from 14 interviews. Reporting followed the Consolidated Criteria for Reporting Qualitative Research.

**Results:**

The core category identified was ‘redefinition of care’, describing a dynamic process in which nurses revised their understanding, adjusted their perspectives, and created new meanings in practice. Key strategies included burden management, exploring new contexts and openness towards uncertainty.

**Discussion:**

The findings suggest that high‐quality care provision extends beyond technical skills, to emotional resilience, interpretive openness and relational flexibility.

**Limitations:**

This study used nurses' retrospective narratives of past cases where recovery progressed. Ongoing cases were not explored.

**Implications:**

Nurses could remain present and responsive even with no immediate solutions, a trait described as ‘negative capability’. Supporting this reflective stance may be key to providing quality care.

**Recommendations:**

Educational approaches potentially strengthening recovery‐oriented care include participatory workshop opportunities that foster pluralistic thinking, dialogue and openness towards uncertainty.

## Accessible Summary

**Originality:**

- We examined the approaches used by psychiatric nurses to support patient recovery in long‐term mental healthcare settings.
- This study drew on interviews with nurses, and particularly the stories that they told, to fill the gap in previous research about how nurses manage the challenges inherent in long‐term mental healthcare.
- We found that some essential skills, competencies, and resources are required to provide sustained care focused on recovery.

**Significance:**

- We found that ‘redefining care’ is a core strategy used by nurses to deliver care focused on recovery in these settings.
- This strategy requires nurses to continually adapt their perspectives and generate new meaning as circumstances change. It also requires nurses to be open‐minded in the face of uncertainty.
- Our findings highlight the importance of flexibility in long‐term mental healthcare nursing.

**Rigour:**

- We used a grounded theory approach and carried out in‐depth interviews with 14 participants. We used both purposive and theoretical sampling until theoretical saturation was reached.
- The researchers held ongoing discussions during the analysis, to check and confirm all findings.
- The participants checked both the interview transcripts and the findings to confirm that they accurately represented their experience.

## 1 Introduction

Over many years, mental health care practitioners and providers have made efforts to move towards a ‘recovery‐oriented’ approach, emphasising personal and unique values and goals rather than the traditional ‘medical model’ characterised by paternalistic care and symptom management (Le Boutillier et al. 2011; Leamy et al. 2011). This change includes long‐term mental health care (Kidd et al. 2014; Killaspy and Priebe 2021; Waldemar et al. 2016). Several initiatives have been described, including systematic care models, defined as organised and structured frameworks of care delivery where procedures, responsibilities, and multidisciplinary collaboration are clearly delineated and managed within institutional systems (Kalisova et al. 2018; Zomer et al. 2024). Other studies have described efforts to reduce seclusion periods using the strengths model (Nagayama et al. 2023), and investigations into the lived experiences of individuals requiring long‐term care (e.g., Ishida and Ishida 2022). However, some studies have highlighted the difficulties involved in successful transition to recovery (Killaspy et al. 2016; Jørgensen and Rendtorff 2018; Jørgensen et al. 2021). Other studies have raised concerns about the quality of care for long‐term inpatients, including limited consideration of their quality of life (Ishida and Ishida 2022) and the restrictive and psychologically distressing effects of controlling and authoritarian care (Bunyan et al. 2017; Zhong et al. 2019). There are also many unmet needs in residential care within community settings (e.g., De Heer‐Wunderink et al. 2012; Slade et al. 2015).

Many professionals are pessimistic about outcomes for users of long‐term mental health services (Caldwell and Jorm 2000; Henderson et al. 2014), and may find it hard to visualise successful treatment outcomes or build relationships in this context (Gaffey et al. 2016; Henderson et al. 2014). Psychiatric and mental health nurses (PMHNs) often have ambiguous professional identities, and struggle to see value in their role (Terry 2020). They may lack time for activities such as listening and talking to people with mental illness (McAndrew et al. 2014). Therefore, nurses in long‐term mental healthcare settings face several sources of uncertainty.

Research on how PMHNs think and practise in ways that support the recovery of people in long‐term mental health care is limited. Some studies have described good practices in long‐term care (Dutta et al. 2016; Nagayama 2024; Salzmann‐Erikson et al. 2016). Salzmann‐Erikson et al. (2016) reported that nurses in forensic psychiatric wards used simple strategies such as ‘sitting on the sofa together with patients’ to better understand them. However, these studies do not explain how nurses develop and implement these good practices.

This study focused on the experiences of PMHNs who have supported recovery in long‐term care settings and used narratives obtained via interviews to describe nurses' abilities, skills, and other resources that enabled them to deliver effective care. We aimed to clarify strategies that allowed PMHNs to engage with long‐term, complex, and uncertain practices and continue reflective thinking over time.

This study had a secondary aim of stimulating discussion in nursing about the concept of ‘negative capability’. This is the ‘capability of being in uncertainties, mysteries, doubts, without any irritable reaching after fact and reason’ (Keats 1817, 71). Keats first used this term in an 1817 letter, where he reflected on Shakespeare's creative imagination and admired the poet's capacity to operate comfortably within uncertainty. This capability is essential for thinking in situations where answers are not forthcoming (Reed 2013), a perspective that Hammer et al. (2012) suggested should be adopted in unpredictable circumstances. Negative capability has been discussed in several disciplines, including psychiatry, psychology, social work and education (Cornish 2011; Coulehan 2017; Hammer et al. 2012; Unterhalter 2017). However, no studies have considered it in nursing. The concept has been interpreted in diverse ways across studies, and there is no agreed‐upon definition. Therefore, this study asked: ‘What abilities enable PMHNs to maintain and develop the care needed to support recovery for people with mental illness in long‐term care?’ We hoped to promote discussion on the applicability of negative capability in nursing practice.

## 2 Methods

### 2.1 Study Design

As mental health care involves a range of complex contexts, we used a qualitative approach, drawing on grounded theory and based on symbolic interactionism. This systematic and inductive methodology is suitable for exploring complex phenomena (Corbin and Strauss 2014). This study was reported in accordance with the Consolidated Criteria for Reporting Qualitative Research checklist (Tong et al. 2007).

### 2.2 Sampling and Recruitment

We used purposeful and theoretical sampling strategies. A recruitment email providing a study overview was sent to the nursing director at a psychiatric hospital in western Japan that has multiple long‐term care wards. After obtaining consent, the nursing director was asked to identify PMHNs who had experience with cases: (1) requiring long‐term care for more than 1 year; (2) where the participant had been substantially involved as a primary nurse; and (3) where the outcome was consistent with previously identified recovery indicators (Liberman et al. 2002; Liberman and Kopelowicz 2005).

Following interviews with four nurses at the first hospital, two additional interviews were conducted at another psychiatric hospital in the same prefecture. This hospital had forensic psychiatric wards, which featured a different care model. Other facilities offered in‐house training about the concept of negative capability, and interviews were extended to two more psychiatric hospitals with long‐term care wards in different prefectures. Both hospitals were in western Japan; one in an urban area (three nurses participated) and one in a rural area (five nurses participated). Data were collected from December 2022 to December 2023. All invited nurses agreed to participate in this study. None withdrew consent during the research process.

Participants were affiliated with either long‐term care wards or community‐based settings, such as visiting nursing stations and daycare facilities. Long‐term care wards are inpatient units that provide ongoing treatment and daily life support for people who require prolonged hospitalisation after completing acute‐phase psychiatric care. Written permission and institutional authorisation were obtained from the ethics committee of the first author's institution (approval no. 22268, dated 20 September 2022) before data collection. Participants received a written explanation of the study and the interview guide. Informed consent was obtained both in writing and verbally, either in person or via video conferencing. The purpose, methods, anticipated benefits and risks, and details of compensation were explained, and participants were informed of their right to decline to participate further at any time. After each interview, participants received a stipend of 2000 yen as remuneration for their time and professional contribution.

### 2.3 Data Collection

Data were collected through semi‐structured interviews conducted either in person or via video conferencing, depending on participant preference. Interviews were held in private rooms at participants' workplaces, with the door closed to prevent anyone overhearing. Participants were informed that all responses would be anonymised, and no identifying information would appear in any publication. Each interview lasted approximately 1 h, during which participants' colleagues might have noticed their temporary absence. However, no details about participation or interview content were disclosed to others. Interviews were audio‐recorded and conducted one‐on‐one, with one interview held per participant. The average interview duration was 72 min (range: 60–88 min).

Participants were asked to share information about a specific episode from their care practice that met the study's inclusion criteria and was particularly memorable or meaningful to them. The interview guide (Appendices S1 and S2) covered (1) an overview of the case, involving either inpatients or clients living in the community; (2) details, intention, and rationale for the care provided; (3) difficulties or challenges faced and how participants experienced and interpreted them; (4) participants' views on reasons for the successful outcomes; (5) factors that enabled ongoing care provision and motivation; and (6) support or influence from others. Demographic information (e.g., age, gender, position and years of experience) was also collected to ensure theoretical variation and contextual understanding among participants. These attributes were considered to be potentially related to nurses' perspectives, interpersonal approaches, and decision‐making in long‐term psychiatric care. Notable verbal expressions and nonverbal behaviours were recorded in memos and used to guide follow‐up questions as appropriate.

The interviews explored both the content of participants' care practices and the thoughts and feelings they experienced while providing care, to understand both what they did and how they thought and felt. They were also asked about factors that shaped their thinking and actions, including experiences beyond the specific case, such as those from nursing practice or daily life. To develop rich narratives, participants were sent the interview guide beforehand to give them time to think about experiences related to the topics. The interviews started with a casual conversation to help establish a relaxed atmosphere.

As the interviews progressed, new questions were added to reflect emerging themes. For example, if personality traits emerged as a key theme, the interviewer explored the relationship between the participant's personality and their practice.

### 2.4 Data Analysis

All interviews were audio‐recorded and transcribed verbatim. The first author repeatedly and thoroughly read the transcripts while taking analytic memos. The transcripts were segmented into units of meaning representing participants' actions and thoughts. Data from 14 participants were then analysed thematically using constant comparison (Corbin and Strauss 2014). In this process, segmented data were constantly compared within and across interviews to identify similarities, differences and relationships among participants' experiences. In the first phase of coding, preliminary concepts were identified that captured the key elements of participants' experiences based on their properties and dimensions. The interrelationships among the concepts were explored simultaneously. This process was iterative and flexible, with constant comparisons used to identify relationships between concepts based on their similarities and differences. A provisional theoretical model was then developed. As the research fields changed, new narratives were compared with the emerging theory. The developing theory was also discussed several times with external researchers and psychiatric mental health nurses who were not directly involved in this study to examine whether new data might alter the emerging theory. The emerging concepts were refined and integrated into higher‐level categories through iterative discussions among the authors. Finally, the findings were integrated into a core category that encompassed the central theme of this study, which led to the development of a theoretical model of long‐term care strategies for PMHNs. Microsoft Excel was used to manage the coding process during the analysis. Data collection continued until theoretical saturation was reached.

### 2.5 Rigour

The first author, a PhD‐qualified university faculty member (male), conceived this study based on research experience in nursing education and practice, and interest in the concept of negative capability. The researcher received interview training during his doctoral studies and had conducted numerous interviews. The researcher met participants for the first time at their interview, but participants were informed in advance about the study's aims and the researcher's interests. To reduce potential bias, care was taken to avoid misunderstandings (e.g., participants offering idealised responses because of the researcher's educational role or perceiving the study as a critique of long‐term psychiatric care). Throughout the interviews, the researcher remained mindful of his responses to ensure participants felt safe to speak freely and did not feel judged.

Credibility, dependability, confirmability, and transferability were used to ensure rigour (Lincoln and Guba 1985). To ensure credibility, this study used constant comparative analysis through reflective and iterative cycles of induction and deduction. This approach maintained a close fit between data and emerging concepts. Participants were also asked to verify that the transcribed interviews accurately reflected their intended meaning, typically within 1–2 months of the interview. Transcripts were not altered and were shared among authors to avoid misinterpretations. Preliminary results were checked by participants, who commented on the representativeness of the categories for actual practice. To ensure dependability, the first author performed open coding, and analytic decisions were discussed among the research team until consensus was reached. An audit trail of analytic decisions was maintained to ensure consistency throughout the coding process. To ensure confirmability, memos for analysis were maintained to distinguish data‐derived interpretations from researchers' assumptions. All coding tables, category definitions, and theoretical diagrams were documented as part of an audit trail, and narrative data were selected to exemplify professional practices. Transferability was supported through theoretical sampling and rich description, allowing readers to assess the applicability of the findings to other contexts.

## 3 Results

### 3.1 Participants' Characteristics

The participants' characteristics are summarised in Table 1. All participants had worked in mental healthcare for much of their professional careers.

**TABLE 1:** Characteristics of participants.

No	Age	Gender	Hospital	Workplace	Years of experience in psychiatric nursing	Years of experience in nursing
1	30s	Male	A	Long‐term care ward	9	9
2	30s	Male	A	General psychiatric ward	16	16
3	40s	Female	A	Long‐term care ward	14	20
4	40s	Male	A	Long‐term care ward	19	24
5	40s	Male	B	Forensic ward (under the Medical Treatment and Supervision Act)	14	17
6	50s	Male	B	Forensic ward	17	17
7	50s	Female	C	Day care or community‐based outpatient services	29	36
8	50s	Female	C	Day care or community‐based outpatient services	12	20
9	50s	Male	C	Long‐term care ward	37	37
10	50s	Female	C	Day care or community‐based outpatient services	31	31
11	50s	Female	C	Day care or community‐based outpatient services	25	30
12	30s	Female	D	General psychiatric ward	15	15
13	50s	Male	D	Long‐term care ward	13	19
14	40s	Male	D	Long‐term care ward	6	6
Mean	46.9 years				18.4	22.2

### 3.2 Categories of Skills and Resources

The analysis yielded nine categories and 39 subcategories (Table 2) representing the skills and resources supporting ongoing long‐term care in psychiatric nursing. Together, they formed a strategic framework. The following sections describe each category/group of categories, concluding with the core category capturing the overall outcome. The final section describes the interrelationships between categories and presents the overarching comprehensive care strategy.

**TABLE 2:** Categories and subcategories of PMHNs' care strategy.

Categories	Subcategories	Definition of subcategories
Routine care	Waiting passively	Waiting for the patient to act or respond while experiencing a sense of uncertainty or anxiety.
	Maintaining patients' daily life	Sustaining the day‐to‐day life of patients by focusing on preventing symptom exacerbation rather than pursuing recovery‐oriented change.
	Sustaining a stagnant relationship	Maintaining a non‐progressive relationship with the patient because of a lack of clear, future‐oriented care planning.
Burdens of long‐term care	Emotional exhaustion	Experiencing emotional turbulence and exhaustion through interactions with the patient.
	Sense of difficulty in thinking about unclear care strategies	Experiencing difficulty because of a lack of clarity about specific care goals or the path towards achieving them.
	Sense of confusion over understanding patients	Experiencing uncertainty or confusion stemming from challenges in interpreting the patient's abilities and traits.
	Sense of inadequacy associated with continuously caring for a person with mental illness without visible change	Experiencing futility or helplessness from continuously observing a person with mental illness who shows little or no change as a result of care.
Burden management	Temporarily distancing from thinking	Intentionally suspending reflective thinking about care to reduce the emotional burden associated with exploring personal practice.
	Temporarily distancing from responsibility	Temporarily attributing the responsibility for care stagnation or failure to external factors to relieve the emotional burden associated with caregiving.
	Assuming that recovery is inherently difficult	Coping with perceived failures in care by focusing on the realistic expectation that recovery may not be easily achievable for that person.
	Sharing emotional burdens with colleagues	Sharing experiences and emotions with colleagues to relieve the emotional burden associated with interaction with patients.
Exploring new contexts	Emergence of an unexpected opportunity for change	When a care opportunity emerges because patients' strength, potential, or will becomes apparent, without direct involvement or intention from nurses.
	Reflecting on care from a distance	Engaging in deliberate reflection on care in a setting or time away from the caregiving context, to gain new insights.
	Communicating outside the context of care	Engaging in non‐care‐related conversations with patients to help build or restore the relationship.
	Valuing the patient's subjective feelings	Prioritising patients' perceptions and feelings as key criteria in care assessment and decision‐making.
	Sharing and understanding the patient's context	Gaining insight into the reasons behind issues by listening to or imagining the contextual background of patients' experience.
	Discovering small signs of success	Experiencing unexpectedly positive feedback from the patient that opens new possibilities for care advancement.
Redefining views of patients	Hoping for recovery	Feeling a sense of hope upon receiving positive responses to care that indicate potential for future improvement.
	Developing a long‐term perspective	Shifting away from expecting immediate results in care planning or changes from the patient, and instead adopting a longer term view.
	Focusing on a smaller scale	Adopting a perspective that focuses on small, everyday changes when larger progress is not readily visible.
	Seeing the big picture	Considering both the care approach and the patient's situation from multiple angles and levels of distance, to explore multiple possibilities.
Expanding the care references	Collecting practical care tips from outside the immediate context	Gathering information or approaches from outside the care context to provide useful clues or strategies for ongoing care.
	Referring to role models	Drawing on the strategies or methods of a specific individual to inform and enhance ongoing care practices.
	Drawing on personal experience	Applying insights and coping strategies gained through other life experiences to inform ongoing care practices.
	Redefining illness	Reinterpreting the illness or psychiatric condition by incorporating perspectives beyond conventional medical knowledge.
Reconstructing the care community	Sharing ideas	Exchanging perspectives and specific strategies about care within the care community.
	Sharing care processes	Ensuring that multiple members of the care community have a shared understanding of the current status and development of the care being provided.
	Creating a place for dialogue	Intentionally setting up opportunities for collaborative discussion about care.
Open‐mindedness about uncertainty	Recognising the reciprocity of care	Becoming aware that providing care can also have a positive emotional or personal impact.
	Accepting ambiguity	Adopting the premise that uncertainty and incomplete understanding are inherent aspects of both patients and the care process.
	Allowing room for failure	Maintaining an attitude that anticipates and accepts both personal failure and failure in colleagues or patients as part of the care process.
	Tolerance for ongoing care	Having a sense of capacity or strategies to sustain repeated actions or persistent reflection over time.
	Curiosity about uncertainty	Demonstrating an active interest or inquisitiveness towards ambiguous or unclear aspects of care and patients.
Redefinition of care	Adapting personal behavioural style to care	Consciously modifying personal tendencies and behavioural orientation to align with the demands or context of caregiving.
	Redefining personal care practice	Engaging in reflective thinking that leads to the reorganisation of care methods or the attribution of new meaning to caregiving.
	Waiting with purpose	Waiting with intention and trust for patients to act, grounded in an understanding of their background and capabilities.
	Struggling alongside patients	Emotionally engaging and collaboratively and persistently exploring possible responses in the absence of clear solutions or methods.
	Standing with the patient	Engaging in communication and negotiation with others on behalf of patients, maintaining respect for their perspectives and emotional needs.

### 3.3 Routine Care

Reasons for long‐term support needs included violence, repeated interpersonal conflicts, limited life skills or behavioural disturbances. These circumstances hindered nurses from identifying clear goals or potential for recovery, resulting in passive, typical care that lacked depth and failed to foster recovery or deeper relationships with service users.

I felt like I wasn't connecting at all. Looking back, maybe our conversations were really superficial…I don't think either of us was opening up emotionally. (Participant 1)

Participants also described difficulty in finding meaning in their nursing practice.

I used to think it was normal to be thanked. When I came here, where “thank you” wasn't the norm, I felt uneasy and restless. I talk with clients, and I help them with things like taking a bath…but still, I sometimes wonder what am I really doing? (Participant 7)

Concern about worsening symptoms or relational deterioration led to overly cautious care approaches, which inadvertently continued routine care. However, this cautiousness provided a foundation for recovery when care plans and goals became clearer.

Even if relationships with patients went bad, we still had to see them every day…I heard others say how hard that was. I thought…that would be hell. I think that's when I started being careful to avoid making things worse. (Participant 6)

### 3.4 Burden of Long‐Term Care/Burden Management

Participants described burdens stemming from continuing to provide routine care and failing to produce visible changes. These burdens led to feelings of frustration, such as the sense of ‘difficulty in thinking about unclear care strategies’ and ‘inadequacy associated with continuously caring for someone without visible change’.

I could understand that he was tired of being told things he didn't agree with. But I still hoped we could achieve something—anything—that would help with his discharge. That pressure…especially early on, was really tough. (Participant 5)

Additional burdens included the ‘sense of confusion over understanding people with mental illness’ and ‘emotional exhaustion’ from interpersonal conflicts.

The things he said were so hard to follow, and he'd get angry out of nowhere. I often didn't understand him or why he was angry. (Participant 9)

As I kept talking with the patient and still couldn't find any clear answers, I gradually started feeling unsure about what I should do. Looking back now, I think I was probably mentally drained. (Participant 2)

Participants described various strategies for managing these burdens, such as stepping away temporarily, sharing the burden with others, or shifting their perspective. The first strategy included ‘temporarily distancing from thinking’ and ‘temporarily distancing from responsibility’, whereby nurses permitted themselves to disengage from care provision briefly, while planning to return.

One of the staff asked me, “Do you want to stop? Who are you doing this for?” I felt so relieved. We decided I'd take a break for a week. I couldn't have made that decision on my own, but hearing it from someone else enabled me to step back. (Participant 7)

The second strategy involved sharing emotional burdens with colleagues or clients/patients themselves.

I try to be honest with them—if it's hard or if it's fun, I tell them. I think that's important…Just saying it out loud helps me organise my thoughts and feel calmer. (Participant 10)

The final strategy emerged from reconsidering the challenges of care, such as stagnation or failure. Participants managed their burdens and motivation by reframing their expectations about recovery.

Trusting people to do things on their own is important, but…if they don't, it is a shock. I had to tell myself not to expect 100%. It's a form of self‐protection. (Participant 1)

These forms of burden management continued after seeing signs of recovery.

### 3.5 Exploring New Contexts

When long‐term care stagnated, participants sought new perspectives to understand their clients by ‘exploring new contexts’. This process involved communicating outside the context of care (i.e., engaging in exchanges not bound by therapeutic purposes or professional roles) or reflecting more deeply about those in their care when away from direct interactions.

It wasn't like I was trying to dig into their symptoms or feelings. I was just enjoying a conversation. In casual chat, sometimes they let slip preferences or what's really on their mind. I wasn't doing it deliberately. (Participant 3)

I make sure I have time alone at least once a day. It's hard to tell others when you are overwhelmed, but you have to do something. So I wrote everything down—what I was feeling, what I was worried about, what I wanted to do. (Participant 10)

Participants reported sincere efforts to understand those for whom they were caring. For example, imagining the background to behaviour could trigger deeper understanding.

She used to call her mum every day and talk about whatever was on her mind. After her mum passed away, she started calling her sister instead—but her sister got cross. I don't think she understood why. I thought maybe she just needed someone to talk to, and perhaps I should try to make more time to talk to her. (Participant 8)

‘Discovering small signs of success’ also sparked hope for the next steps in care.

When I heard he gave a speech at the ward event, I was like, “Wait, he can do that?” That made me think—if he can do that, maybe he can also step out of his room. It gave me hope. (Participant 1)

At times, these discoveries occurred unexpectedly, independent of the nurse's intention or involvement. For example, a nurse described someone initially rejecting their suggestion but being influenced by someone else nearby—an unplanned interaction that facilitated care progression.

He got cross with me, but then someone else at the same table said, “I can only have a snack during this time too,” and he just settled down. It wasn't planned—it just happened. I was surprised, but realised he could go along with the group when the timing was right. (Participant 3)

### 3.6 Redefining Views of Clients/Expanding the Care References/Reconstructing the Care Community

After ‘exploring new contexts’, nurses discovered new aspects of their clients, and in turn, changed themselves. These changes manifested as: ‘redefining views of people with mental illness’, ‘expanding the care references’ and ‘reconstructing the care community’. Participants emphasised the importance of taking a long‐term perspective, based on the premise that the care process is inherently long‐term.

I heard once that one day of treatment in general medicine is like a week in psychiatry. I think that's true—you have to have a longer‐term mindset. You can't expect rapid change. We need to be okay with watching and waiting…. (Participant 5)

Nurses also learned to change their perspective. For example, focusing on very small changes in their clients, or adopting a more distanced, macro‐level viewpoint to broaden interpretive possibilities.

Just from their faces when they walk in, or how they respond when I greet them, I can tell if something's going on, or if I should check in. I think picking up on those small changes is really important. (Participant 8)

I often tell young nurses this. When you're about to say or do something, I think it's important to take a step back and look at the situation first. In psychiatric settings, acting on impulse can be risky. I try to encourage others to pause and think it through…more objectively and imagine what might happen as a result. (Participant 1)

Expanding their perspective also helped nurses cultivate hope by noticing the potential for change in their clients. In ‘expanding the care references’, participants adopted skills and interpretations from role models and drew on their own life experiences such as parenting experiences.

As a parent, I had a lot of inner conflict. However, when I thought about it more broadly, I realised that my child had their own life and world. My patients do too. Their illness is part of their life, and we need to relate to them as individuals. That changed how I saw things. (Participant 8)

Other participants described how experiences of loss deepened their empathy.

Nurses made efforts to accumulate care skills through training or observation, even if those skills were not immediately relevant. As their references expanded, their understanding of illness diversified, meaning they redefined mental illness, and sometimes interpreted symptoms in a more affirming or integrated way.

When a patient shouts loudly, some staff interpret that as a worsening symptom. Others see it as stress relief. I think I'm more in that second group now. I try to see behaviour in a positive light…. (Participant 2)

I used to focus entirely on improving symptoms—meds, injections, and so on. But then I started to realise how much of the struggle happens in real life…The real journey starts after discharge. (Participant 10)

In ‘reconstructing the care community’, participants became more intentional about sharing care ideas and progress to create spaces for discussion.

I didn't know what to do, and neither did the discharge support team…We asked the client's permission to discuss things together. I told them I couldn't do it alone and wanted everyone's input. They agreed. That really helped. (Participant 7)

### 3.7 Open‐Mindedness About Uncertainty

The burdens of long‐term care, efforts to expand their thinking, and the transformation of care appeared to be grounded in what participants described as ‘open‐mindedness about uncertainty’. This meant being able to continue working despite failure or unclear outcomes, and being open to ambiguity.

I think it's about understanding human beings—not just psychiatric patients. People make mistakes. They do things that don't always make sense to others, even if there's some internal logic. (Participant 14)

Participants described accepting uncertainties, including the ambiguous link between care and outcomes, differences in staff approaches or the role of contingency and timing.

Sometimes, it's just a matter of time. Sometimes it's luck. There are so many factors involved. (Participant 7)

Open‐mindedness was not necessarily viewed as a special talent. Many participants described it as embedded in their way of working.

Just facing the person in front of me and continuing to say, “This is what you need” doesn't feel especially hard to me. It just feels natural. (Participant 2)

This attitude towards uncertainty was linked to a desire to explore. Some participants described this as a personality trait associated with a drive to understand.

I'm the kind of person who wants to know more. If something goes well, I don't leave it at that—I want to understand why. I've always been like that. (Participant 1)

Participants noted that they sometimes received valuable input from their clients. These unexpected experiences helped them to better understand unpredictable clients.

He sometimes got really excited by his hallucinations and acted out, but I also felt this huge sense of warmth and openness from him—this ability to accept others. That really stayed with me. (Participant 9)

Open‐mindedness about uncertainty was seen as both an ability acquired through experience and an inherent personality trait.

### 3.8 Core Category: Redefinition of Care

The emerging core category was the ‘redefinition of care’. During routine care, participants lacked a clear sense of goals or meaning in their nursing practice. However, through the processes described, they discovered new perspectives and values in their practice. When routines were repeated or progress appeared limited, this redefinition allowed them to accept their burden of care and actively and intentionally engage in waiting.

It felt like our hearts connected—we were sharing the same pain. I doubt she really understood what I was trying to say, but in that moment, I truly felt like a psychiatric nurse for the first time. Before that, I was just instructing them or doing medical tasks—approaching them from a place of authority. (Participant 9)

This redefinition altered how participants viewed persistent symptoms. When they recognised trustworthy or admirable qualities in the individual, they became more accepting of phenomena such as fixed delusions.

He seemed to believe in his delusion, but then he told me he'd continue with his treatment. That made me think—maybe deep down, he still had a grasp on reality. And hearing him say that made me feel, “Maybe this is okay.” (Participant 6)

Waiting also gained new meaning. Despite the future being unclear, the emergence of hope helped to justify waiting without seeing it as stagnation.

I still can't see what's coming, but I want to walk alongside them, through the good and bad. It's their life, after all. They should be the ones choosing, not us. Our job is to support them. (Participant 10)

This shift in perceptions about waiting had secondary effects. It supported listening and improved collaboration with clients.

First, I just listen and try to empathise with how hard it must be to come to day care carrying all those feelings. Just saying, “Yeah, I hear you”—that's the first step. (Participant 8)

This redefinition of care was strengthened through reflective practices such as writing case reports.

Writing up the case gave me a chance to think about how I was interacting with them. Through that process, something I'd been doing without thinking started to feel more conscious. (Participant 7)

Despite care having been redefined, participants reported that recovery sometimes stalled. This suggested that redefining care is a cyclical and ongoing process not a one‐off event.

For a while, everything went smoothly. He had goals, and we were helping him reach them, but then he suddenly stopped expressing what he wanted. I was worried that if we pushed too hard, it might stress him out or trigger a relapse, so I backed off a bit. It felt like we were back to square one, but I thought…it's better than risking his stability’. (Participant 1)

### 3.9 Overview of Nursing Care Strategy

**FIGURE 1:**
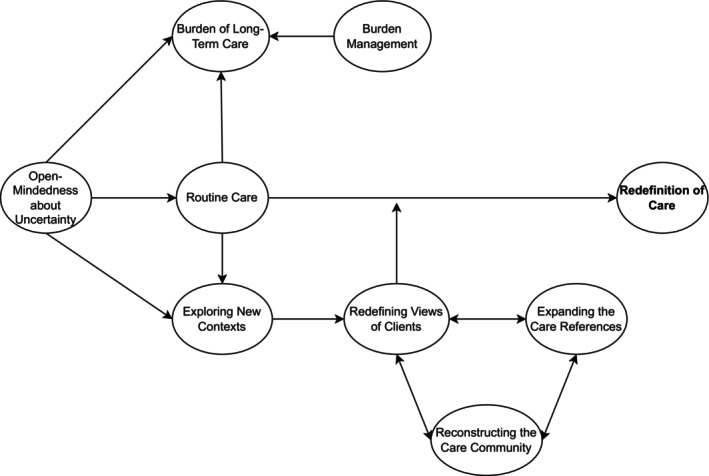
The interrelationships among the categories of the PMHNs' skills and resources in the long‐term care.

Nurses' long‐term care strategies were characterised by their efforts to redefine care during situations where goals and meaning were difficult to discern, such as during the stagnation of routine care. These strategies were sustained by managing the emotional burden of care, expanding their perspectives, and reconstructing their working environments. Figure 1 shows the interrelationships among categories in this strategy framework.

## 4 Discussion

PMHNs promote recovery in long‐term, complex and uncertain care settings by shifting their perspectives on care and those for whom they are caring, and redefining the meaning of care. Previous research showed that long‐term care is distressing for people with mental illness (Ishida and Ishida 2022; Kiss et al. 2021; Zhong et al. 2019; Zomer et al. 2024). Bunyan et al. (2017) reported that long‐term care in psychiatric rehabilitation wards imposed a heavy burden on nurses. Our study highlights this major burden across all psychiatric wards. Burden management played a critical role in the continuation of care despite an unclear future. Earlier studies emphasised the role of external support in alleviating nurses' burden (Dutta et al. 2016), but our findings suggest that internal strategies (e.g., cognitive reframing) are just as important.

Our category of ‘redefining views of clients’ was consistent with previous findings. Salzmann‐Erikson et al. (2016) and Dutta et al. (2016) described practices encompassing ‘getting to know the person (patient)’, which reflected a deepening of our understanding of individuals. We found this understanding was not solely constructed in nurse–client interactions, but supported by changes in nurses themselves. Our category ‘exploring new contexts’ showed that change can be initiated accidentally. Although McKenna et al. (2014) emphasised the importance and effectiveness of guidelines for recovery, our findings suggest the need for daily educational and training practices that cultivate flexibility in responding to unexpected developments in psychiatric care. Studies of medical students have reported the use of educational approaches such as workshops using art‐based discussions to explore multiple interpretations (Bentwich and Gilbey 2017) and simulation exercises incorporating clinical uncertainty (Scott et al. 2020). Adapting these experiential learning opportunities for nursing students may enhance their ability to understand and respond flexibly to the unpredictability inherent in psychiatric care.

Sustained capacity and adaptability in unpredictable situations appeared to be underpinned by ‘open‐mindedness about uncertainty’. Keats (1817) coined the term ‘negative capability’ to describe Shakespeare's creative ability. The qualities that nurses in this study demonstrated appeared consistent with that concept. One participant described their thinking style as ‘I tend to question things a lot’, which resonates with the defining feature of negative capability—an effort to transcend closed intellectual systems (Hammer et al. 2012). Unlike previous interpretations, we found that nurses' persistence and creativity were sustained and enhanced by interactions with others. This may reflect the unique professional characteristics of nursing.

To date, the concept of negative capability has primarily been explored in professions with one‐on‐one, continuous engagement with clients such as psychiatry (e.g., Reed 2013). However, the dynamic nature of nursing with its shifts in working hours and assigned patients/clients, may shape a unique form of negative capability. Supportive colleagues may be crucial for nurses to promote recovery without becoming jaded, and enable them to continue to foster creative approaches to care. Reed (2013) emphasised the importance of ‘finding opportunities for brief pauses’ to sustain negative capability. It may be equally vital in long‐term care to cultivate a human environment that supports these pauses.

The core category ‘redefinition of care’ showed that as service users changed and recovered, nurses shifted their perspectives and attitudes towards care. Repper et al. (1994) noted that long‐term support for individuals with mental illness required a long‐term outlook and skills to adapt flexibly to clients. Other studies described how nurses can support transformation in clients (Gee et al. 2017; Nagayama 2024; Salzmann‐Erikson et al. 2016; Whitley et al. 2009). However, the redefinition we identified suggests that these practices are accompanied by changes in nurses. This redefinition of care was a cyclical and dynamic process. It highlights the need for a dialectical approach, accepting the lack of visible change in people with mental illness and the importance of waiting, yet maintaining a self‐reflective stance and continuing to seek new ways of thinking and acting. In the longer term, educational support may be necessary to sustain such reflective thinking, and cultivate environments where these attitudes are supported.

## 5 Study Limitations

This study used PMHNs' retrospective narratives, reflecting on past cases where they had reached a stage of personal meaning‐making. We did not consider cases where recovery did not progress, or nurses were still navigating difficulties. Further studies are needed to explore such cases using a similar analytical lens. The concept of recovery in this study reflected nurses' perspectives and may not be consistent with the views of service users or objective indicators of recovery.

## 6 Conclusion

This study shows how PMHNs promote recovery in long‐term, uncertain care settings by redefining their roles, perspectives and care strategies. Central to this process is a capacity for tolerating ambiguity and an ability to continuously reflect and adapt. Educational efforts that cultivate these qualities may improve the sustainability and quality of psychiatric nursing.

## 7 Relevance for Clinical Practice

To continue providing care under uncertain conditions, PMHNs must possess both persistence and creativity—two seemingly opposing capacities. To advance the long‐term use of recovery‐oriented care, educational support is needed to foster multifaceted perspectives and interpretive flexibility. The dissemination of the concept of negative capability may help support these developments.

## Funding

This work was supported by JSPS KAKENHI (Grant Number 21K10710).

## Ethics Statement

This study was approved by the University of Osaka Hospital Ethical Review Board (No. 22268).

## Conflicts of Interest

The authors declare no conflicts of interest.

## Supporting information


**Appendix S1:** jpm70071‐sup‐0001‐AppendixS1.docx.

**Appendix S2:** jpm70071‐sup‐0002‐AppendixS2.docx.

## Data Availability

The data that support the findings of this study are available on request from the corresponding author. The data are not publicly available due to privacy or ethical restrictions.
