# Supplementary material for: Skills and Resources of Psychiatric Mental Health Nurses to Support a Long and Uncertain Recovery Journey: A Grounded Theory Approach
Source: J Psychiatr Ment Health Nurs. 2025 Dec 2;33(1):140–52. doi: 10.1111/jpm.70071 (PMC12770798; doi:10.1111/jpm.70071)
Supplement: Supplementary file 1 — Appendix S1: jpm70071‐sup‐0001‐AppendixS1.docx. [file JPM-33-140-s001.docx]

Interview Guide

Interview Procedure

1. Greeting the participant

2. Explanation of the study overview and ethical considerations

3. Explanation of the consent form and withdrawal form

4. Completion of the consent form after confirming willingness to participate

5. Start of the interview

6. Questions regarding demographic information

7. Questions about a case the participant was continuously involved with for over one year (case overview, nursing practices, thoughts and feelings, etc. No fixed question order; start with topics that are easiest for the participant to talk about, such as overall impressions. See details below.)

8. Confirmation of any additional comments or statements the participant wishes to retract

9. Thanking the participant and concluding the interview

10. Presentation of honorarium

11. At a later date, participants are asked to review the interview transcript and confirm whether they wish to withdraw or revise any statements

Details of questions

Demographic Information

• Age

• Gender

• Qualifications (e.g., Certified Nurse, Specialist Nurse)

• Work experience and years (at current facility, at other facilities, in other departments, other occupational experience)

Regarding the Case

• Background of the service user: age, gender, diagnosis, reasons for long-term care needs, course of the case

• Goals of nursing practice and their development

• Description and progression of nursing practices provided

• Challenges or difficulties encountered during nursing practice

• Thoughts and feelings during nursing practice

• Perceived reasons for achieving nursing goals or outcomes

• Level of motivation during practice and how it changed over time

• Reflections on why the participant was able to continue providing care

• Support received from others during nursing practice

• Current reflections and evaluation of the nursing practice

Regarding the Participant’s Own Negative Capability

• What kind of negative capability the participant believes is necessary as a psychiatric nurse

• How the participant believes they developed their own negative capability as a psychiatric nurse
