# Supplementary material for: Skills and Resources of Psychiatric Mental Health Nurses to Support a Long and Uncertain Recovery Journey: A Grounded Theory Approach
Source: J Psychiatr Ment Health Nurs. 2025 Dec 2;33(1):140–52. doi: 10.1111/jpm.70071 (PMC12770798; doi:10.1111/jpm.70071)
Supplement: Supplementary file 2 — Appendix S2: jpm70071‐sup‐0002‐AppendixS2.docx. [file JPM-33-140-s002.docx]

**「精神科看護師におけるNegative Capability概念の構築」**

**インタビューガイド**

1. 研究目的

精神科看護師の看護実践に内在する不確かさと向き合う力であるNegative Capabilityを、インタビュー調査を通して探索し概念構築を目指す

1. インタビュー日時

研究対象者に、自身の勤務に支障のない日時をご提示頂き、調整を行う

場所

施設の看護部長もしくは管理者に対して、対象者のプライバシーが保障される個室あるいは個室に準ずる環境の提供を依頼する。インタビュー調査の場所については、研究対象者から別途希望があった場合は、その希望に沿った場所でインタビューを行う。（※感染症対策として、対象者の希望があった場合はオンラインでのインタビューを行う。）

1. インタビューにあたり配慮すること

- 資料1を用いて、本研究の目的、方法、倫理的配慮を説明した上で、研究対象者の自由意思に基づいて同意が得られているかの確認を行う。いったん同意が得られた場合であっても、辞退可能であることを保証する。
- 同意が得られた場合、1 時間程度の時間を頂くこと、面接内容は IC レコーダーにて録音することに関して了承を得る。録音内容は研究者以外が聞くことはないこと、録音内容を全て転記した後は、破棄することを説明する。
- 手指消毒、マスクの着用、定期的な換気、使用後の物品の消毒といった感染対策を徹底した上で実施する。
- オンラインインタビューを希望された場合、セキュリティの高いソフトを使用し、実施する。

インタビューの流れ

1. あいさつ
2. 研究概要および倫理的配慮に関する説明
3. 同意書・同意撤回書の説明
4. 調査参加意思確認後に同意書の記入
5. 調査開始
6. 基本属性に関する質問
7. 1年以上継続的に関わった事例についての質問（事例の概要、その時行ったこと、考えたこと、感じたことなど。設問の順番は特に設けないが、実習の印象など研究対象者が話しやすいものから聞いていく。設問の詳細は下記参照）
8. 言い忘れ、撤回したい発言の有無について確認
9. 調査終了のあいさつ
10. 謝品を渡す
11. 後日、研究対象者には逐語録を確認してもらい、再度発言等の撤回がないか確認する。

調査項目

- 基本属性

年齢、性別、資格の有無（専門/認定看護師など）、勤務経験とその年数（当該施設、当該施設外、他科での経験、その他職業経験）

- 事例について

事例患者の背景：年齢、性別、診断名、支援が長期に必要だった理由、事例の経過

看護実践の目標とその変遷

どのような看護実践を行なっていたか、またその経過や変遷

看護実践の中で困難や問題に感じていたこと

看護実践にあたりどのようなことを考えていたか、感じていたか

看護目標やアウトカムを達成できた理由をどう考えているか

看護実践の中で自らのモチベーションはどうだったか、もしくはどう変化したか

看護実践を継続できた理由をどのように考えているか

看護実践の中で周囲からどのようなサポートがあったか

看護実践を今どう考え、評価しているか

- 自分自身のNegative Capbilityについて

精神科看護師としての自分自身のNegative Capbilityとはどのようなものが必要と考えるか

精神科看護師としての自分自身のNegative Capbilityはどのように獲得されたと考えているか
